# Highly tumoricidal efficiency of non-oxidized MXene-Ti_3_C_2_Tx quantum dots on human uveal melanoma

**DOI:** 10.3389/fbioe.2022.1028470

**Published:** 2022-10-06

**Authors:** Huankai Zhang, Xuesong Li, Pan You, Xian Song, Qian Fan, Xutang Tao, Yi Qu

**Affiliations:** ^1^ Department of Ophthalmology, Shandong Provincial Qianfoshan Hospital, Shandong University, Jinan, China; ^2^ State Key Laboratory of Crystal Materials, Shandong University, Jinan, China; ^3^ Department of Cell Biology, School of Basic Medical Sciences, Shandong University, Jinan, China

**Keywords:** uveal melanoma, non-oxidized MXene-Ti_3_C_2_Tx quantum dots, reactive oxygen species, ferroptosis, mitophagy

## Abstract

Uveal melanoma (UM) is a highly malignant intraocular tumor with poor prognosis. Current topical ophthalmic therapies purpose to conserve the eye and useful vision. Due to the risks and limited clinical benefits, the topical treatments of UM remain challenging and complex. In this study, newly developed non-oxidized MXene-Ti_3_C_2_Tx quantum dots (NMQDs-Ti_3_C_2_Tx) are proposed for UM treatment. Surprisingly, NMQDs-Ti_3_C_2_Tx shows significant tumor-killing effects on UM cells in a dose-dependent manner and causes severe necrosis near the injection site on the xenograft UM tumor model. Moreover, NMQDs-Ti_3_C_2_Tx exhibits excellent biocompatibility with normal retina pigment epithelium (RPE) cells and does not cause any damage in C57BL/6 mice eyes. Mechanistically, NMQDs-Ti_3_C_2_Tx inhibits the proliferation, invasion, and migration of UM cells *via* its desirable reactive oxygen species (ROS) generation ability, which causes lipid peroxidation and mitophagy, triggering cell ferroptosis. Furthermore, NMQDs-Ti_3_C_2_Tx is detected accumulating in autolysosomes which exacerbates cell death. This work provides new light on the topical treatment of UM.

## 1 Introduction

Uveal melanoma (UM) is the most common intraocular malignancy that occurs in adults (Mallone et al., 2020). Currently, the first-line topical treatments targeting UM aimed to conserve the eye and useful vision are radiotherapy, surgery, transpupillary thermotherapy (TTT) and photodynamic therapy (PDT). Radiotherapy, including plaque brachytherapy and radiation, is the most common eye-conserving therapy and has achieved certain efficacy in UM therapy (Jager et al., 2020). However, these treatments need surgeons to place plaques or place fiducial markers; the side effects of radiotherapy, such as damage to the neighboring normal tissues, neovascular glaucoma, retinal detachment, or cataract, have plagued clinicians for years (Bianciotto et al., 2010). Surgery, including local resection, enucleation, and exenteration, is always employed when the tumor is unsuitable for radiotherapy (Jager et al., 2020). Local resection could probably maximize the removal of neoplasm while keeping the integrity of the eye as possible, but even then, vision is affected by different degrees. Enucleation and exenteration could affect the facial appearance of patients, which further impacts their quality of life. TTT and PDT were initially designed as alternatives to radiotherapy; however, these therapies show limited clinical benefit while carrying several risks (Pereira et al., 2013). Recently, AU-011, a novel kind of PDT, has been approved by Food and Drug Administration (FDA) as an orphan drug targeting UM, but the tumor-killing effect of AU-011 is limited if the tumor has an anterior location and could not be irradiated entirely (Kines et al., 2018). Hence, topical ophthalmic therapies for UM aimed to conserve the eye and useful vision are still needed.

Over the past few decades, due to the fast-growing area of nanotechnology, Chemodynamic therapy (CDT), one kind of novel tumor therapeutic strategy, has achieved breakthroughs. Iron-based nanocatalysts, which could release ferrous ions, are the most common catalysts for CDT. Other nanocatalysts, such as Cu^1+^ and Mn^2+,^ also reveal the desirable catalytic ability (Nie et al., 2019; Wang et al., 2020; Lin et al., 2021). They could initiate the Fenton reaction and catalyze hydrogen peroxide (H_2_O_2_) into hydroxyl radical (•OH), which exerts powerful toxic effects on tumor cells (Tang et al., 2019). Thus, CDT based on nanocatalysts exhibits great potential applications in the ocular treatment of UM. However, conventional CDT has several shortcomings. Due to the weakly acidic tumor microenvironment (TME), the efficiency of the Fenton reaction is relatively low, which cannot generate enough reactive oxygen species (ROS) to kill tumor cells (Ranji-Burachaloo et al., 2018). Besides that, the potential toxicity risk of nanoparticles might be dosage-dependent (Hu et al., 2018). To avoid potential side effects, more efficacious and safer nanoparticles for CDT need to be explored.

As reported previously (Li et al., 2020), we have successfully prepared non-oxidized MXene-Ti_3_C_2_Tx quantum dots (NMQDs-Ti_3_C_2_Tx), a new kind of Ti-based nanocatalysts with excellent dispersion and stability. Surprisingly, NMQDs-Ti_3_C_2_Tx exhibits highly efficient tumor-killing effects on cervical and breast cancer cells and shows excellent biocompatibility. Therefore, the characteristics of NMQDs-Ti_3_C_2_Tx inspire us that the new Ti-based material may have great potential to be applied to UM as a kind of agent *in situ*.

**FIGURE 1:**
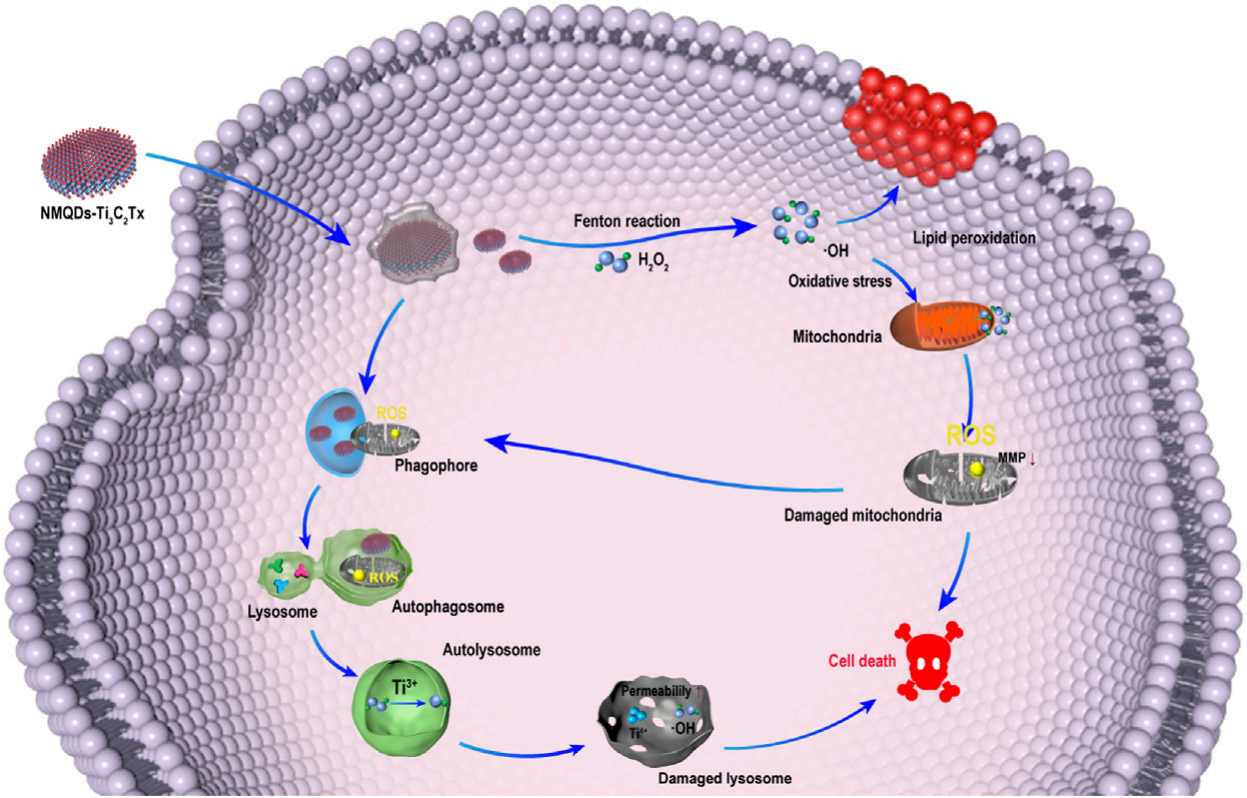
Schematic illustrating the antitumor pathway of NMQDs-Ti_3_C_2_Tx.

In this study, we focus on evaluating the tumoricidal efficacy of NMQDs-Ti_3_C_2_Tx on UM cells and UM xenograft mouse models. As expected, the performance of NMQDs-Ti_3_C_2_Tx in UM cells is incredible. NMQDs-Ti_3_C_2_Tx shows a robust tumor-killing capacity in UM cells. NMQDs-Ti_3_C_2_Tx could significantly inhibit the activity of UM cells and show no protumor effect even below 100 μg ml^−1^. More importantly, NMQDs-Ti_3_C_2_Tx only has toxicity to tumor cells near the injection site in UM xenograft mouse models, which is very suitable for ocular topical therapies. Biocompatibility of NMQDs-Ti_3_C_2_Tx is explored on retinal pigment epithelium (RPE) cells and in normal eyes of C57BL/6 mice. No apparent toxicity is observed. Mechanistically, NMQDs-Ti_3_C_2_Tx could induce a large amount of •OH in UM cells, resulting in lipid peroxidation and mitochondrial dysfunction. Meanwhile, NMQDs-Ti_3_C_2_Tx is detected accumulating in autolysosomes, and the ROS reaction in autolysosomes could destruct the autolysosomes and further accelerate cell death (Figure 1). As a result, a low dosage of NMQDs-Ti_3_C_2_Tx could have the potential for topical therapy on UM.

## 2 Materials and methods

### 2.1 The synthesis and characterization of NMQDs-Ti_3_C_2_Tx

NMQDs-Ti_3_C_2_Tx was synthesized using a micro-explosion method described previously (Li et al., 2020). The MXene-Ti_3_C_2_Tx and NMQDs-Ti_3_C_2_Tx were characterized using a Hitachi S-4800 field-emission SEM system and a JEOL JEM 2100F transmission electron microscope.

### 2.2 Cell lines and cell culture

Human RPE cell line, UM cell line C918, and UM cell line MUM-2B were purchased from the Chinese Academy of Sciences Cell Bank (Shanghai, China). RPE cells were cultured in Dulbecco’s modified Eagle’s medium (DMEM, Gibco) with 10% fetal bovine serum (FBS, Gibco). UM cells were cultured in RPMI-1640 (Gibco, United States) with or without 10% FBS. All cell lines were grown at 37°C and 5% CO_2_.

### 2.3 Cell viability assay

Cell-counting kit-8(CCK-8) assay (Beyotime, China) was used to examine the effect of NMQDs-Ti_3_C_2_Tx on normal and UM cell viability. Before incubation with the indicated concentrations of NMQDs-Ti_3_C_2_Tx for different incubation times, cells were seeded into a 96-well plate at 3.0 × 10^3^ cells per well and cultured for 12 h for cell attachment. Considering the absorbance of NMQDs-Ti_3_C_2_Tx itself, the fresh medium was replaced before adding 10 μL of CCK-8 solution per well. After 3 h of incubation at 37°C and 5% CO_2_, the absorbance at 450 nm was measured.

### 2.4 Cell live/dead staining

Cells were seeded into a 6-well plate at 7.0 × 10^5^ cells per well and cultured for 12 h for cell attachment before incubation with the indicated concentrations NMQDs-Ti_3_C_2_Tx for 6 h. Then, Cells were washed gently once in phosphate-buffered saline (PBS) before Live/Dead (Abbkin, China) assay. Finally, a fluorescence microscope (Olympus IX 73 fluorescence microscope, Japan) was used to capture the images.

### 2.5 Colony formation assay

Cells were seeded into a 6-well plate at a density of 300 cells per well. Then, cells were incubated with different concentrations of NMQDs-Ti_3_C_2_Tx for approximately 2 weeks. The indicated concentrations of NMQDs-Ti_3_C_2_Tx were added every 3 days during this period. Once the colonies were visible to the naked eye, the cells were fixed using 4% paraformaldehyde, and then 0.1% crystal violet solution was used to stain the cells. The number of cell colonies was counted using a microscope and ImageJ in the end.

### 2.6 Cell invasion assay and cell wound-healing assay

The invasion assays were performed using a Transwell BD Matrigel (Corning, United States). The cells previously incubated with different concentrations of NMQDs-Ti_3_C_2_Tx for 6 h were seeded in upper chambers in a serum-free medium. At the same time, 800 μL cultured medium with 20% FBS was supplemented in lower chambers. Before adding UM cells, diluted Matrigel (ratio: 1:8) (Corning Costar, United States) was added to the upper chambers. After 24  h, cells were stained with 0.1% crystal violet. A microscope was used to capture the images.

The cells previously incubated with different concentrations of NMQDs-Ti_3_C_2_Tx for 6 h were seeded into a 6-well plate at a density of 7.0×10^5^ cells per well. Cells were scratched with a sterile 200-μL pipette tip after adhering to the wall. Cells were then cultured with different concentrations of NMQDs-Ti_3_C_2_Tx. At 0 and 12 h, the images of wounds were captured.

### 2.7 Measurement of ROS generation, lipid hydroperoxide generation, mitochondrial membrane potential (MMP) alteration, Malondialdehyde (MDA), and Glutathione (GSH)

Cells were seeded into a 6-well plate at a density of 7.0×10^5^ cells per well. After 12 h, cells were incubated with different concentrations of NMQDs-Ti_3_C_2_Tx for 6 h. The cells were then incubated with H2DCFDA (Beyotime, China), C11-BODIPY (MkBio, China), or Tetramethylrhodamine (TMRM, Sigma, United States) solution according to the instruction. According to the manufacturer’s protocol, MDA content was measured using an MDA assay kit (Abbkin, China), and GSH content was measured using a GSH/GSSG assay kit (Beyotime, China).

### 2.8 Real-time PCR

According to the manufacturer’s protocol, the total RNA from UM cells was isolated using the Fastagen (Feijie Biotech, China). RNA then underwent reverse transcription using the NeuScript II 1st strand cDNA synthesis kit (Vazyme, China). Then, the SYBR Green (CWBIO, China) real-time PCR (RT-PCR) was analyzed on Roche LightCycler 96 system. The relative RNA expression of PTGS2, SLC7A11 and LC3 were normalized using the RNA expression of GAPDH. The specific primers applied for RT-PCR reaction are shown in (Supplementary Table S1).

### 2.9 Electron microscopy

After 6 h stimulation of NMQDs-Ti_3_C_2_Tx, UM cells were harvested and fixed with the electron microscope fixation liquid at 4°C for storage. 1% osmic acid and 0.1 M phosphate buffer (PH7.4) mixture were used for 2 h post-fixation. UM cells were then dehydrated with different concentrations of alcohol and then dehydrated with 100% acetone twice. Next, cells were infiltrated with acetone/812 embedding agent and embedded in a pure 812 embedding agent. Then, UM cells were placed at 60°C for 48 h. Next step, UM cell samples were chopped into 60–80 nm sections and stained with 2% uranyl acetate saturated alcohol solution for 8 min in the dark. 2.6% Lead citrate was used to avoid CO2 staining. Finally, the copper mesh slices were dried at room temperature overnight in a copper mesh box. Images were observed under a transmission electron microscope.

### 2.10 Lysosomes and mitochondrion colocalization

UM cells were seeded in a 6-well plate containing slides and then cultured with NMQDs-Ti_3_C_2_Tx (100 g ml^−1^) for 1 h. The Lyso-Tracker Red and Mito-Tracker Red (Beyotime, China) were used to stain the lysosomes and mitochondrion of UM cells, respectively. Finally, UM cells were observed on a fluorescence microscope (Olympus IX 73 fluorescence microscope, Japan).

### 2.11 Animal studies

All experimental procedures were approved by the Laboratory Animal Ethical and Welfare Committee of Shandong University Cheeloo College of Medicine (Shandong, China). The permission number of animal experiment ethical approval is 20158.

Intraocular injection mouse model: Six C57 BL/6 mice were randomly divided into two groups. Mice in the control group received unilateral intraocular injections of 1 μL saline. Mice in the experimental group received unilateral intraocular injections of 1 μL NMQDs-Ti_3_C_2_Tx (2 μg L^−1^). After 3 days, all mice were sacrificed under deep anesthesia, and the eyes were excised for further characterization.

UM xenograft mouse model: Eight BALB/c nude mice (6-week-old female) were subcutaneously injected with 100 μl of 5 × 10^6^ C918 cells to the right shoulder. The mice were randomly divided into two groups (n = 4) for antitumoral studies when the tumor volume reached 50–100 mm^3^. The tumor-bearing mice were treated with 1) saline and 2) 10 mg kg^−1^ NMQDs-Ti_3_C_2_Tx, *via* intra-tumoral administration. Tumor volume and body weight were recorded every 2 days. The tumor volume was measured using the formula: tumor volume (mm^3^) = (length × width^2^)/2. On the eighth day after the first administration, xenograft tumors were excised for further characterization.

### 2.12 Statistical analysis

Data are presented as mean ± standard error of the mean (error bars). Every experiment was repeated at least three times independently. The differences between two groups were evaluated using Student’s t-test, whereas the statistical analysis of multiple groups was performed using the one-way ANOVA. Statistics were calculated using GraphPad Prism 7. Significant differences are indicated as *p < 0.05, **p < 0.005, ***p < 0.0005, ****p < 0.0001.

## 3 Results and discussion

### 3.1 Synthesis of NMQDs-Ti_3_C_2_Tx

**FIGURE 2:**
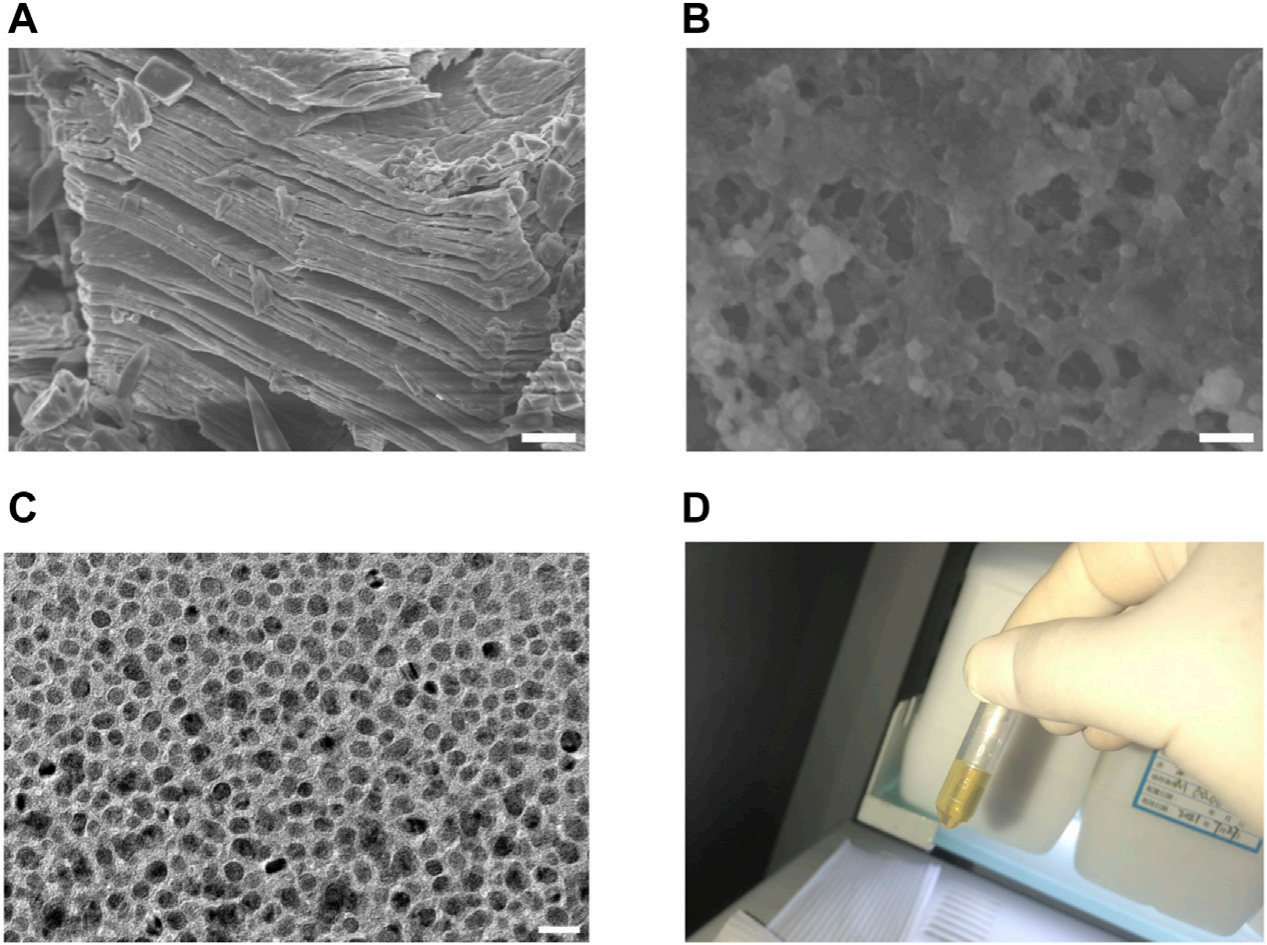
The preparation and characterization of NMQDs-Ti_3_C_2_Tx **(A)** SEM image of MXene-Ti_3_C_2_Tx. Scale bar: 2 μm. **(B)** SEM image of MXene-Ti_3_C_2_Tx after microexplosion. Scale bar: 200 nm **(C)** TEM image of NMQDs-Ti_3_C_2_Tx. Scale bar: 20 nm. **(D)** the aqueous solution of NMQDs-Ti_3_C_2_Tx.

We first prepared MXene-Ti_3_C_2_Tx, the raw material of NMQDs-Ti_3_C_2_Tx, through etching Ti3AlC2. The MXene-Ti_3_C_2_Tx has a typical accordion-like microstructure (Figure 2A). Then, NMQDs-Ti_3_C_2_Tx was obtained by using the micro explosion method as previously reported by our group (Li et al., 2020). We inserted liquid nitrogen (L-N_2_) into Ti_3_C_2_Tx layers and subsequently loaded with boiling deionized water. Drastic temperature changes caused micro-explosion. Finally, the pale-yellow powders of NMQDs-Ti_3_C_2_Tx were obtained from aqueous dispersion by freeze-drying. Moth-eaten cavities caused by micro explosion demonstrated that NMQDs-Ti_3_C_2_Tx was successfully prepared (Figure 2B). Through the transmission electron microscopic (TEM), we could find NMQDs-Ti_3_C_2_Tx exhibits a regular disc shape (Figure 2C). And the aqueous solution of NMQDs-Ti_3_C_2_Tx is pale yellow (Figure 2D).

### 3.2 NMQDs-Ti_3_C_2_Tx inhibits UM cell proliferation and clonogenic potential

**FIGURE 3:**
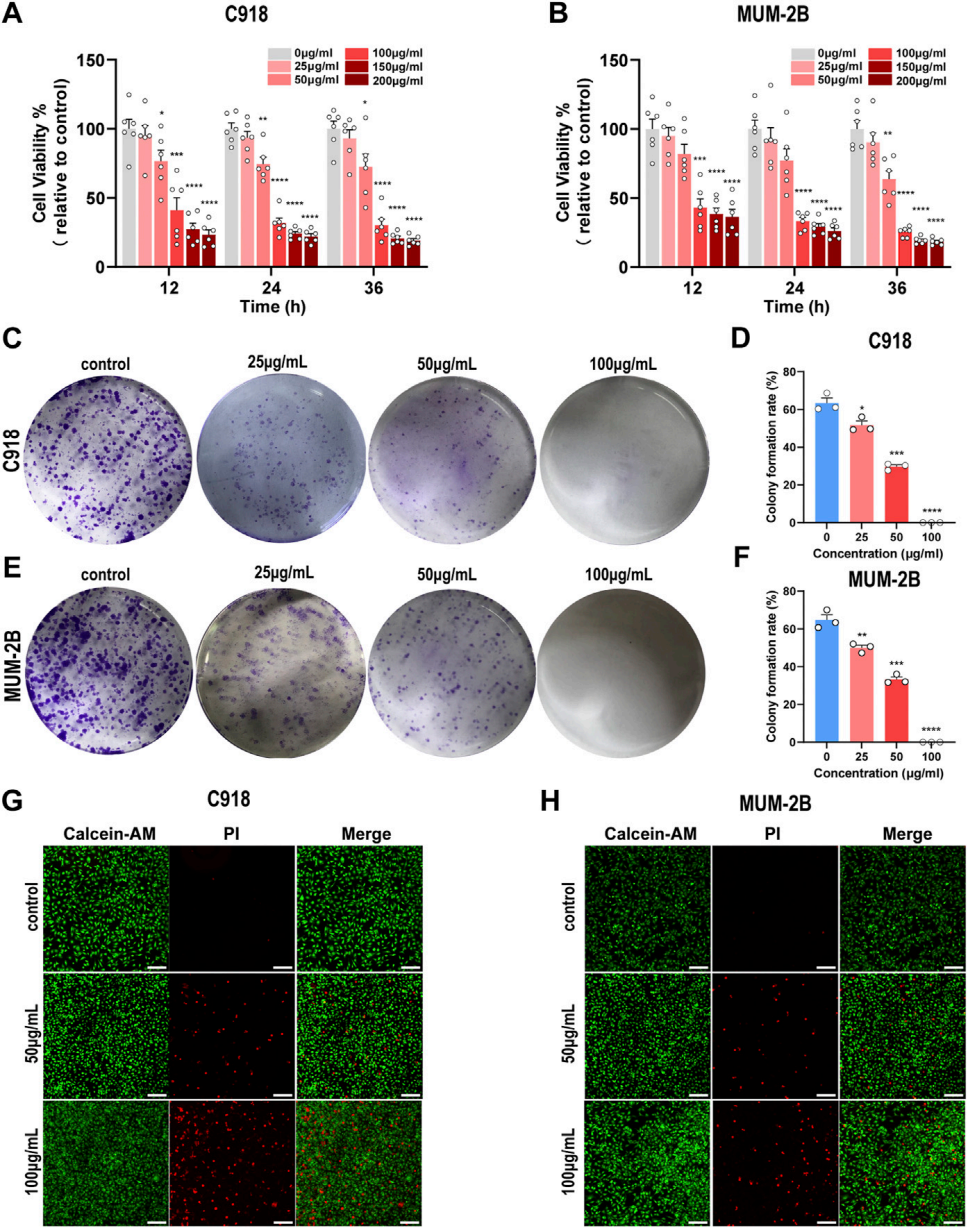
NMQDs-Ti_3_C_2_Tx inhibits UM cell proliferation and clone formation **(A and B)** The CCK-8 assay of C918 and Mum-2B cells cultured with different concentrations of NMQDs-Ti_3_C_2_Tx for 12, 24, and 36 h (n = 6; *p < 0.05, **p < 0.005, ***p < 0.0005, ****p < 0.0001) **(C and E)** Colony formation assay results. C918 and Mum-2B UM cells were cultured with different concentrations of NMQDs-Ti_3_C_2_Tx for 12 days **(D and F)** The clone formation rate results were counted according to the graph C and E (n = 3; *p < 0.05, **p < 0.005, ***p < 0.0005, ****p < 0.0001). **(G and H)** Live/dead staining results. The live cells were stained with Calcein- AM, and the dead cells were stained with PI. Scale bar: 200 μm. All above data are presented as the mean ± SEM.

To assess the cytotoxicity of NMQDs-Ti_3_C_2_Tx, Two UM cell lines C918 and Mum-2B were treated with different concentrations (0, 25, 50, 100, 150, and 200 μg ml^−1^) of NMQDs-Ti_3_C_2_Tx for different times (12, 24 and 36 h). Effects of NMQDs-Ti_3_C_2_Tx on cell proliferation were analyzed by a CCK-8 assay. As shown in Figures 3A,B, the proliferation viability of UM cells was decreased in a dose-dependent manner. For C918 cells, the IC50 values of NMQDs-Ti_3_C_2_Tx at 12 h, 24 h, and 36 h were 69.80 μg ml^−1^, 63.37 μg ml^−1^, and 63.15 μg ml^−1^, respectively; while for MUM-2B cells, the values were 64.36 μg ml^−1^, 63.28 μg ml^−1^, and 55.94 μg ml^−1^, respectively. Low cytotoxicity for normal cells is one of the essential properties of nanocatalysts in tumor detection and therapy, especially in the human eye, a highly specialized organ of vision. Hence, normal retina pigment epithelium (RPE) cell line was used as a control. As shown in (Supplementary Figure S1), there was no significant effect on the normal RPE cell viability cultured with NMQDs-Ti_3_C_2_Tx after different incubation times, even at a high concentration (200  μg ml^−1^). These results illustrated that NMQDs-Ti_3_C_2_Tx have high toxicity for UM cells, not for healthy ocular cells.

To further investigate the ability of NMQDs-Ti_3_C_2_Tx on the viability of UM cells, we also measured the reproductive viability of single cells by plate colony formation assays, another experiment reflecting the potential malignant properties of tumor cells. In C918 and MUM-2B cell lines, the number and the size of colonies were mildly decreased in the presence of 25 μg ml^−1^ NMQDs-Ti_3_C_2_Tx; at the concentration of 50 μg ml^−1^, the number and the size of colonies were significantly decreased. In addition, there was no colony formation at the concentration of 100  μg ml^−1^ and 200 μg ml^−1^ (Figures 3C–F; Supplementary Figure S2). However, in the CCK-8 assay, we observed no statistically significant difference between 0  μg ml^−1^ and 25  μg ml^−1^. We considered that the long-term stimulation of NMQDs-Ti_3_C_2_Tx for UM cells in plate colony formation assays led to different outcomes compared with the CCK-8 assay.

Moreover, C918 and MUM cells were subjected to live/dead staining, where viable cells were stained with Calcein-AM (green) and dead cells were stained with PI (red). After being stimulated with NMQDs-Ti_3_C_2_Tx for 6 h, few dead cells were observed when cultured with NMQDs-Ti_3_C_2_Tx at 50 μg ml^−1^, while a small number of dead cells were observed when cultured with NMQDs-Ti_3_C_2_Tx at 100 μg ml^−1^ (Figures 3G,H). Hence, to avoid cell death-related effects, UM cells were cultured with indicated concentrations (50 and 100 μg ml^−1^) of NMQDs-Ti_3_C_2_Tx for 6 h in subsequent experiments.

### 3.3 NMQDs-Ti_3_C_2_Tx inhibits UM cell invasion and migration

It is well known that tumor invasion and migration are the two essential features of tumor metastasis. Upon detachment from the primary site, invasion and migration of tumor cells into surrounding microvasculature of the lymphatic and blood vessels start, eventually resulting in tumor colonization of distant organs (Polacheck et al., 2013). Up to 50% of patients with UM will have metastases. Once metastases develop, the prognosis becomes poor. Although 5-year survival rates of UM patients have not improved, early intervention for UM could efficiently conserve the eye and useful vision with additional potential for improving long-term survival (Kines et al., 2018). Hence, we wonder whether NMQDs-Ti_3_C_2_Tx has the potential to suppress UM cell invasion and migration.

**FIGURE 4:**
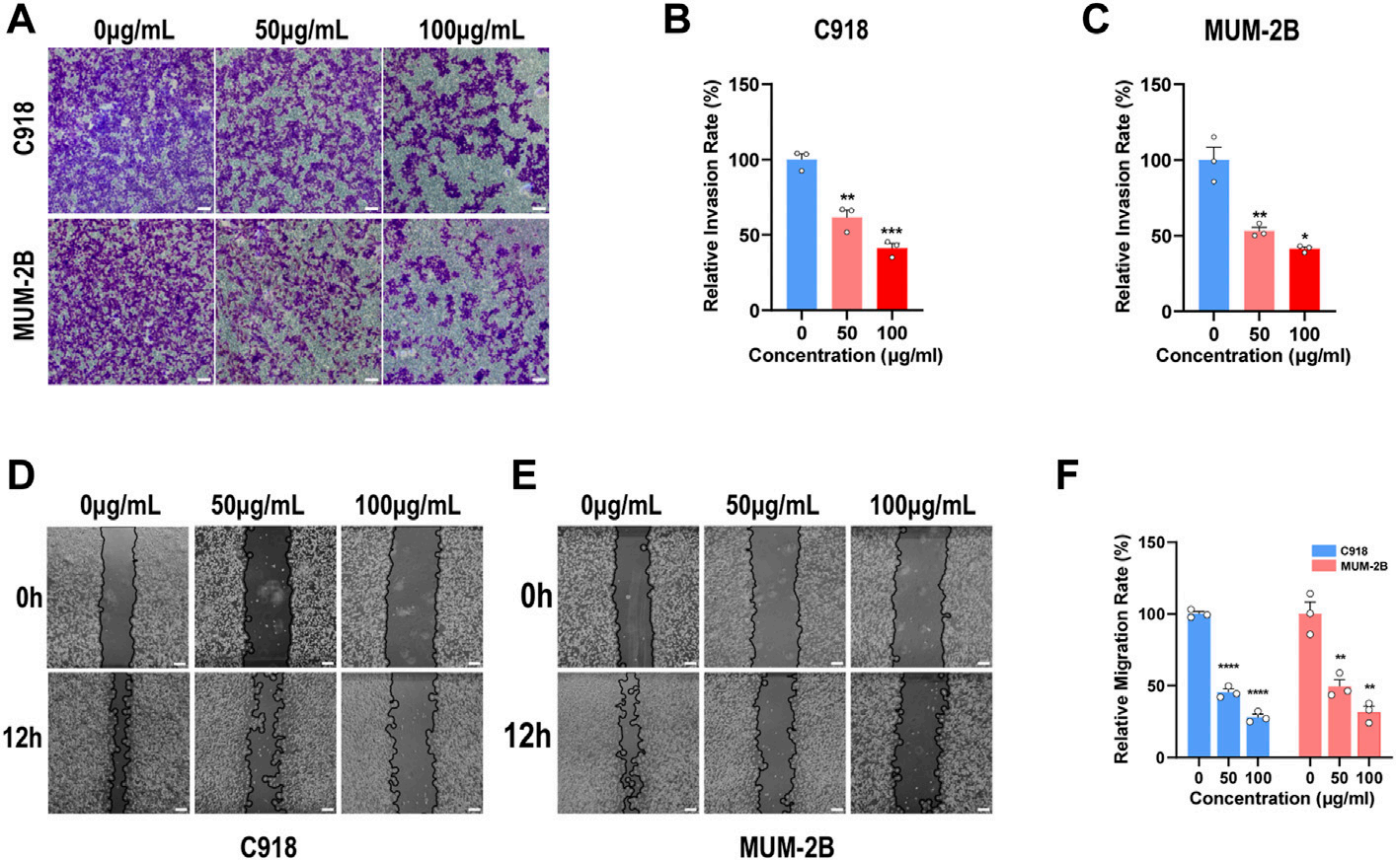
NMQDs-Ti_3_C_2_Tx inhibits UM cell invasion and migration **(A,B and C)** The transwell assay of the UM cells cultured with different concentrations of NMQDs-Ti_3_C_2_Tx. Scale bar: 200 μm. **(D,E and F)** The wound healing assay of the UM cells cultured with different concentrations of NMQDs-Ti_3_C_2_Tx. Scale bar: 200 μm. These assays were analyzed and calculated using ImageJ (n = 3, *p < 0.05, **p < 0.005, ***p < 0.0005, ****p < 0.0001). The above data are presented as the mean ± SEM.

A transwell invasion assay was employed to explore the UM cell invasion capacity, and a wound-healing assay was used to investigate the UM cell migration capacity. In order to guarantee the correct results, UM cells were cultured with indicated concentrations of NMQDs-Ti_3_C_2_Tx for 6 h before the experiments. Following the increased concentration of NMQDs-Ti_3_C_2_Tx in UM cell lines, the results of the Transwell assay revealed that the number of migratory cells was significantly decreased (Figures 4A–C). The invasion ability of UM cells decreases by 50% as the concentration of NMQDs-Ti_3_C_2_Tx increases to 100 μg ml^−1^. Similarly, the wound healing assay results also illustrated that NMQDs-Ti_3_C_2_Tx significantly inhibited the invasive ability of UM cells (Figures 4D–F). Accordingly, the marked ability of NMQDs-Ti_3_C_2_Tx to suppress UM cell invasion and migration may achieve an additional benefit in the course of treatment for UM.

### 3.4 NMQDs-Ti_3_C_2_Tx causes lipid peroxidation and triggers ferroptosis in UM cells

We further investigate the molecular mechanisms of NMQDs-Ti_3_C_2_Tx in UM cells. Our previous study has illustrated that NMQDs-Ti_3_C_2_Tx is a strong reducing agent, and it can generate the highly reactive •OH by readily reacting with H_2_O_2_(Li et al., 2020). Within the cytoplasm, •OH is the most reactive ROS and is an initiator of lipid peroxidation (Sies and Jones, 2020). •OH could attack the polyunsaturated fatty acids (PUFAs) of lipid membranes and induce lipid peroxides generation, a well-established mechanism of cellular injury (Del Rio et al., 2005). Thus, we investigated whether ROS and lipid peroxides would be generated in UM cells cultured with NMQDs-Ti_3_C_2_Tx.

**FIGURE 5:**
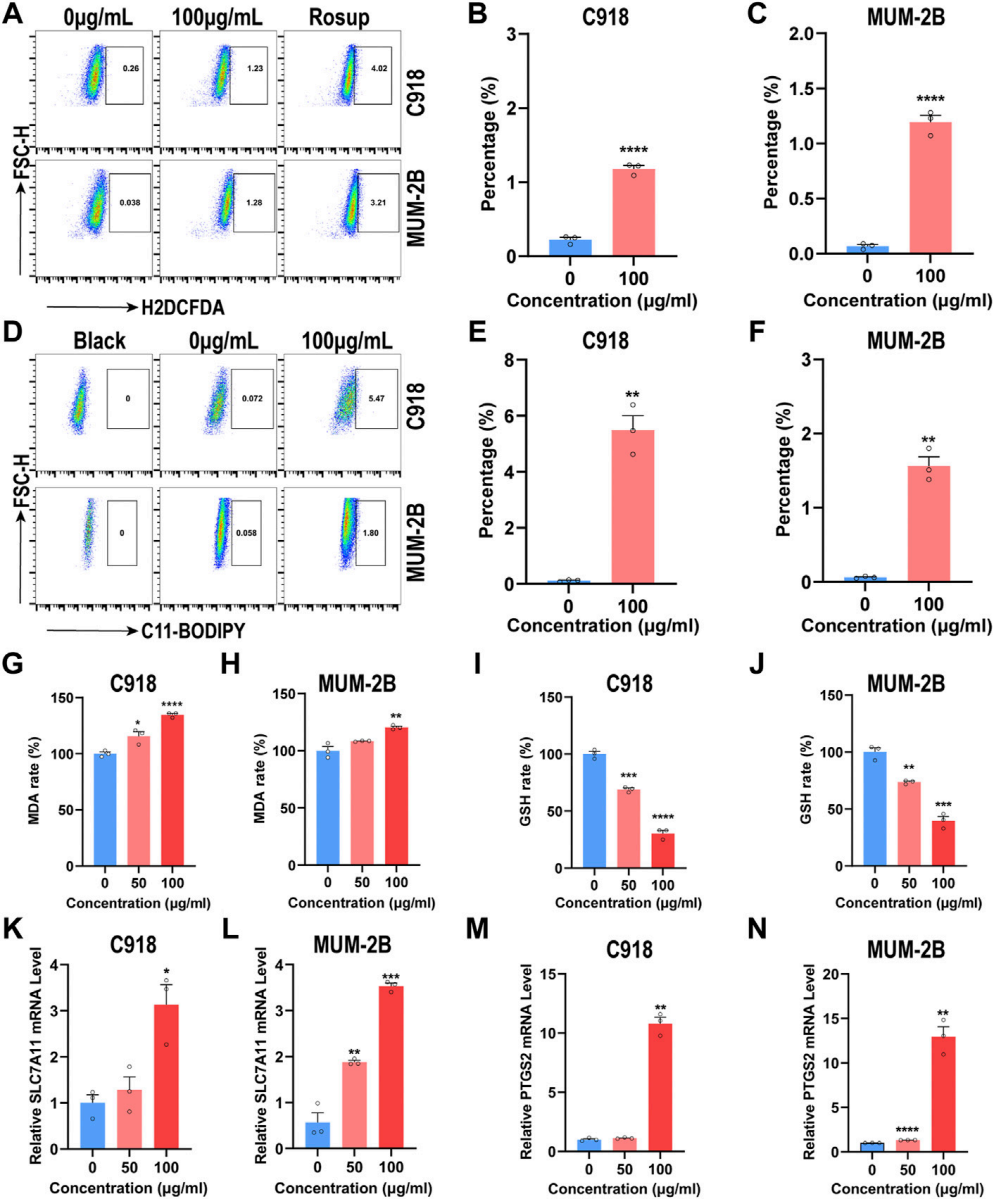
NMQDs-Ti_3_C_2_Tx causes ROS accumulation and induces ferroptosis in UM cells **(A,B and C)** The ROS levels in UM cells incubated with different concentrations of NMQDs-Ti_3_C_2_Tx were determined by flow cytometry coupled with H2DCFDA staining. Rosup, a ROS inducer, was used as the positive control (n = 3, ****p < 0.0001, compared with the control group) **(D,E and F)** The levels of lipid peroxides in UM cells treated with different concentrations of NMQDs-Ti_3_C_2_Tx were determined by flow cytometry coupled with C11-BODIPY staining (n = 3, **p < 0.005) **(G and H)** The MDA levels of UM cells after 6 h of incubation with 100  μg ml^−1^ of NMQDs-Ti_3_C_2_Tx (n = 3; *p < 0.05, **p < 0.005, ****p < 0.0001) **(I and J)** The GSH levels of UM cells after 6 h of incubation with 100  μg ml^−1^ of NMQDs-Ti_3_C_2_Tx (n = 3; **p < 0.005, ***p < 0.0005, ****p < 0.0001) **(K,L)** The SLC7A11 RNA levels of UM cells after 6 h of stimulation with NMQDs-Ti_3_C_2_Tx. GAPDH was used as a loading control (n = 3; *p < 0.05, **p < 0.005, ***p < 0.0005, ****p < 0.0001) **(M,N)** The PTGS2 RNA levels of UM cells after 6 h of stimulation with NMQDs-Ti_3_C_2_Tx. GAPDH was used as a loading control (n = 3; *p < 0.05, **p < 0.005, ***p < 0.0005, ****p < 0.0001). The above data are presented as the mean ± SEM.

To demonstrate whether •OH is induced in the cytoplasm in UM cells incubated with NMQDs-Ti_3_C_2_Tx, we used the ROS fluorescence probe 2′,7′-dichlorofluorescein diacetate (H2DCFDA) to evaluate the intracellular •OH generation (Li et al., 2020) and analyzed through Fluorescence Activating Cell Sorter (FACS). As shown in Figures 5A–C, after 6 h of NMQDs-Ti_3_C_2_Tx (100 μg ml^−1^) stimulation, the levels of intracellular ROS markedly increased in UM cells. In contrast, the ROS production was not significantly altered when the probe H2DCFDA was added to RPE cells cultured with NMQDs-Ti_3_C_2_Tx (Supplementary Figure S3), which indicated the presence of NMQDs-Ti_3_C_2_Tx did not cause a significant increase of •OH in RPE cells. These suggested that ROS production contributes to UM cell death. Carbon dots (Cdots) are another kind of nanomaterial to exert tumoricidal effects by the production of ROS. Cdots exhibit a pro-tumorigenic role for UM at concentrations below 100 μg ml^−1^ (Ding et al., 2021). For NMQDs-Ti_3_C_2_Tx, we do not need to be concerned about the double-edged role of ROS induced by NMQDs-Ti_3_C_2_Tx in UM cells.

We then investigated whether UM cells exhibited lipid peroxidation incubated with NMQDs-Ti_3_C_2_Tx. The probe C11-BODIPY was used to measure lipid peroxidation. After stimulation of NMQDs-Ti_3_C_2_Tx, very similar findings were observed. We found an apparent accumulation of lipid peroxides in UM cells, not in RPE cells (Figures 5D–F; Supplementary Figure S4). The presence of MDA, a marker of lipid peroxidation, portends the disruption of membranous structures in cells (Simon et al., 2020). We evaluated the MDA levels in UM cells using an MDA assay kit. As expected, the levels of MDA elevated as the concentration of NMQDs-Ti_3_C_2_Tx was increased in UM cells (Figures 5G,H). Ferroptosis is a unique cell death program driven by lipid peroxidation (Dixon et al., 2012; Verma et al., 2020). The accumulation of Lipid peroxides and MDA indicated that NMQDs-Ti_3_C_2_Tx triggered ferroptosis in UM cells.

In tumor cells, the unusually high concentrations of ROS result in metabolic dysregulation. Naturally, adaptive antioxidant mechanisms would be subsequently developed (Yang et al., 2019). GSH is a substrate for GSH peroxidase 4 (GPX4) that protects cells against ROS, and it can combine with lipid peroxides to form oxidized GSH (GSSG). Usually, the intracellular GSH concentration in a cancer cell is higher than in a normal cell (Jung et al., 2013). But excess ROS would decrease the GSH level, triggering cell death. For this reason, we assessed the GSH levels in UM cells. After incubation with NMQDs-Ti_3_C_2_Tx, the intracellular GSH concentrations in UM cells are lower than that in control groups (Figures 5I,J). Cystine is the essential precursor of GSH synthesis, and System xc−, a cystine/glutamate exchange transporter, leads to the uptake of cystine into the cell cytosol (Sato et al., 1999). When the intracellular GSH shortage is present, the transporter can be upregulated (Sasaki et al., 2002). Subunit solute carrier family seven member 11(SLC7A11) is the catalytic subunit of System xc−. Thus, we appraised the System xc− activity through measurement of SLC7A11. Incubated with NMQDs-Ti_3_C_2_Tx, the RNA levels of SLC7A11 were upregulated (Figures 5K,L). This result showed negative feedback on GSH. We also investigated the mRNA level of PTGS2 which is one of the recognized ferroptosis-related genes. After stimulation of NMQDs-Ti_3_C_2_Tx, the mRNA levels of PTGS2 were upregulated (Figures 5M, N). These results demonstrate that NMQDs-Ti_3_C_2_Tx induces excessive production of •OH and consequently causes lipid peroxidation. Finally, Overwhelming lipid peroxidation triggers ferroptosis in UM cells.

### 3.5 NMQDs-Ti_3_C_2_Tx induces mitophagy and autolysosome destruction in UM cells

Although •OH is the most reactive ROS, it reacts with biomolecules directly at the site of its generation (Galaris et al., 2019). Excessive ROS would destroy mitochondria, causing a decrease in MMP, consequently leading to cell death (Cheng et al., 2019). The mitochondrion is one crucial source of endogenously produced H_2_O_2_(Dickinson et al., 2010). We wonder whether mitochondrion is affected by the •OH induced by the NMQDs-Ti_3_C_2_Tx.

**FIGURE 6:**
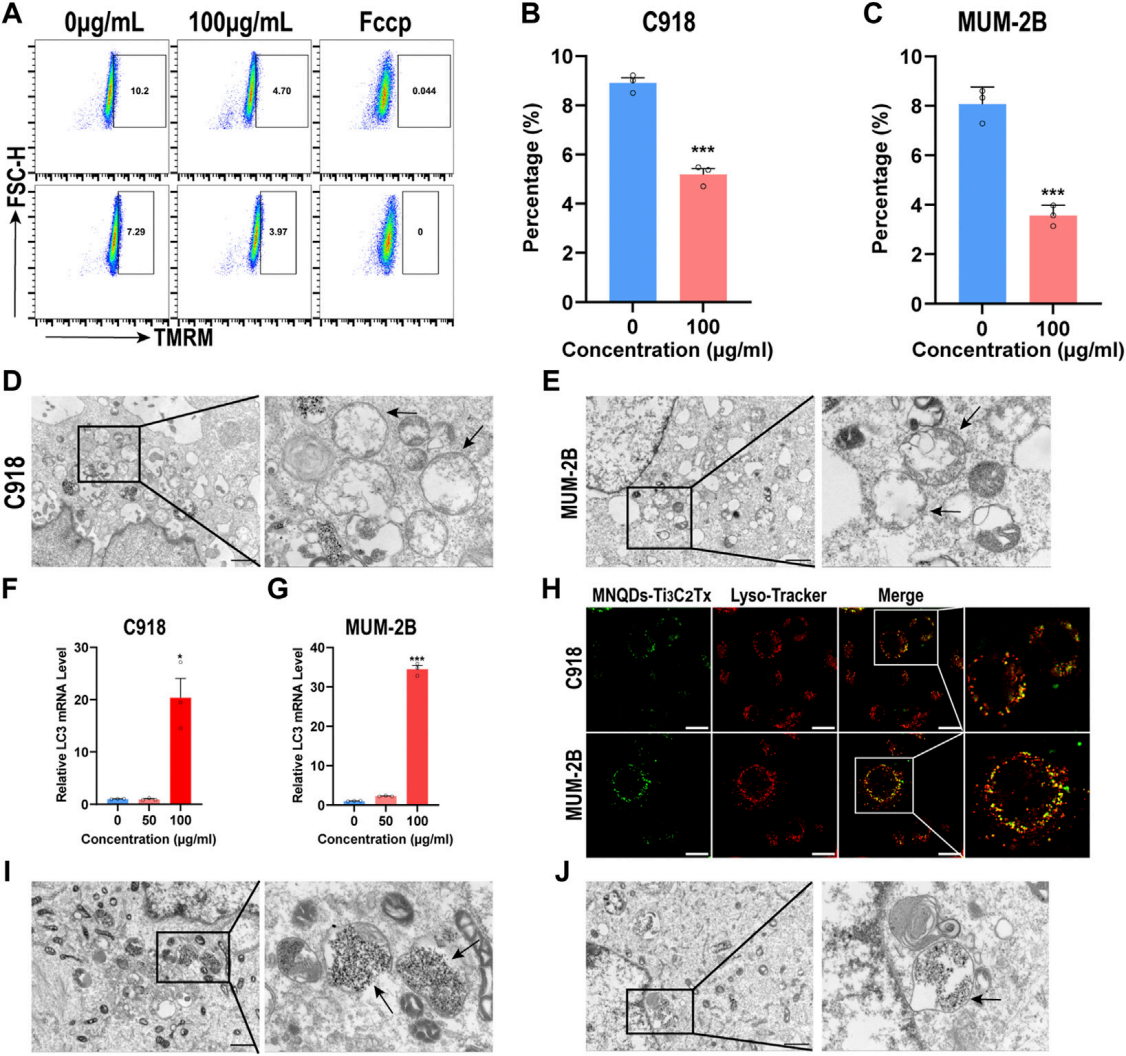
NMQDs-Ti_3_C_2_Tx induces mitophagy and lysosome destruction in UM cells **(A,B and C)** The levels of MMP in UM cells treated with different concentrations of NMQDs-Ti_3_C_2_Tx were determined using flow cytometry coupled with TMRM staining. Fccp was used as the negative control with low MMP (n = 3, ***p < 0.0005) **(D and E)** Bio-TEM image and partial enlarged images of UM cells after treated with 100  μg ml^−1^ of NMQDs-Ti_3_C_2_Tx. The black arrows point to mitochondrial edema. Scale bar: 10 μm. **(F and G)** The LC3 RNA levels of UM cells after 6 h of stimulation with 100  μg ml^−1^ of NMQDs-Ti_3_C_2_Tx. GAPDH was used as a loading control (n = 3; *p < 0.05, ***p < 0.0005) **(H)** Colocalization of NMQDs-Ti_3_C_2_Tx and lysosomes in UM cell lines. Scale bar: 20 μm. **(I and J)** Bio-TEM and partial enlarged images of UM cells after treated with NMQDs-Ti_3_C_2_Tx. The black arrows indicated autolysosomes. Scale bar: 10 μm.

We used a plasmid to track mitochondrion to prove this, and we found that NMQDs-Ti_3_C_2_Tx is colocalized with mitochondrion in UM cells (Supplementary Figure S5). Mitochondrial fluorescence dye TMRM was used to determine the MMP in a flow cytometer. As expected, MMP decreased significantly after NMQDs-Ti_3_C_2_Tx addition (Figures 6A–C). In contrast, MMP in RPE cells showed no significant change (Supplementary Figure S6). We also investigated the ultrastructure of mitochondria by electron microscopy. Through the view of the electron microscope, we observed many mitochondrial edemata in the UM cells incubated with NMQDs-Ti_3_C_2_Tx, not in normal UM cells, and the structures of mitochondria were severely damaged (Figures 6D,E; and Supplementary Figures S7,S8). These results indicate that accumulated NMQDs-Ti_3_C_2_Tx in UM cells leads to mitochondrial dysfunction, resulting in cell death.

Lysosomes are acidic organelles specialized in the degradation and recycling of macromolecules (Tai et al., 2020). Mitochondrial autophagy, a specialized type of autophagy, is a conserved lysosome-dependent process that plays a vital role in mitochondrial quality control and removing fragmented mitochondria (Ashrafi and Schwarz, 2013). Multiple causes cause mitochondrial dysfunction, and damaged mitochondria are specifically recognized and sequestered into autophagosomes that fuse with lysosomes to degrade mitochondria. In UM cells, NMQDs-Ti_3_C_2_Tx attacks mitochondria, resulting in mitochondria dysfunction. We hypothesized that mitophagy might occur in UM cells incubated with NMQDs-Ti_3_C_2_Tx. The LC3 subfamily is considered an autophagy marker (Weidberg et al., 2010). The RNA levels of LC3 were upregulated cultured with NMQDs-Ti_3_C_2_Tx in UM cells (Figures 6F,G), which proved the induction of autophagy. We also observed the ultrastructure of UM cells using electron microscopy. Under the electron microscope, we observed autolysosome formation. But we only found damaged mitochondria and black granules in autolysosomes in the therapeutic groups (Figures 6I,J and Supplementary Figures S9,S10). Extracellular macromolecules enter the cell by endocytosis and eventually terminate with lysosomal degradation. We further investigated whether NMQDs-Ti_3_C_2_Tx could accumulate in lysosomes. We used fluorescent probes to track lysosomes to prove this, and we found that NMQDs-Ti_3_C_2_Tx is colocalized with lysosomes (Figure 6H). In this way, the black granules are the result of NMQDs-Ti_3_C_2_Tx accumulation in autolysosomes. The lysosome is an acidic organelle, which provides the opportunity for NMQDs-Ti_3_C_2_Tx to induce Fenton reaction, thus leading to plasma membrane integrity disruption of the autolysosomes (Figures 6I,J). As a result of the disruption, lysosomal hydrolases are released into the cytosol, resulting in lysosomal cell death with necrotic features (Aits and Jaattela, 2013).

### 3.6 Activity and biocompatibility of NMQDs-Ti_3_C_2_Tx *In vivo*

**FIGURE 7:**
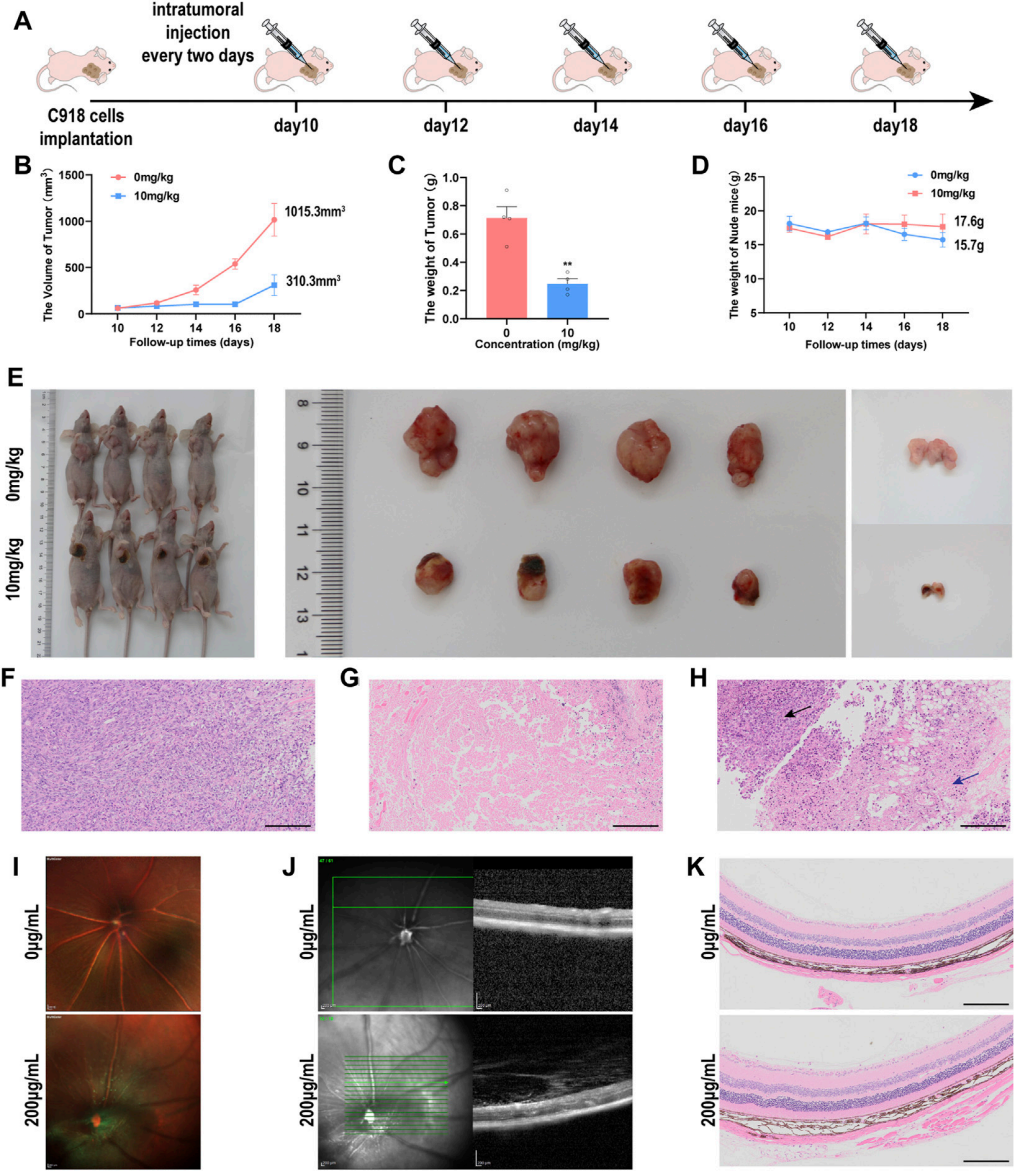
The multi-mode imaging *in vivo* **(A)** Timeline of the tumor-bearing nude mice experiment. **(B)** the tumor sizes of the UM tumor-bearing mice during the treatment **(C)** Tumor weights of the UM tumor-bearing mice at the end of the treatment (n = 4, **p < 0.005) **(D)** The body weights of nude mice bearing UM tumor xenografts during the treatment. **(E)** Images of the sizes of xenograft UM tumors during NMQDs-Ti_3_C_2_Tx treatment **(F)** H&E staining of the tumors from control group. Scale bar, 200 μm. **(G)** H&E staining of the tumors from the therapeutic group. Scale bar, 200 μm **(H)** H&E staining of the tumors from the demarcation of therapeutic group. The black arrow points to the normal tumor tissue, and the blue arrow points to necrosis. Scale bar, 200 μm. **(I)** Ocular fundus images of experimental mice. Scale bar: 200 μm **(J)** OCT images of the mice retina. Scale bar: 200 μm. **(K)** H&E staining of the retina from the mice. Scale bar: 200 μm.

Given the excellent tumor-killing efficacy of NMQDs-Ti_3_C_2_Tx *in vitro*, we continued to test its therapeutic efficacy *in vivo*. C918 cells were injected into the right axilla of the mice to create the UM subcutaneous tumor model. When the tumor grew to 50–100 mm^3^ in volume, NMQDs-Ti_3_C_2_Tx (10 mg kg^−1^, the therapeutic group) and the same volume of saline (the control group) were injected intratumorally every 2 days. A total of five injections were performed (Figure 7A). During the period, the sizes of the tumors and the weights of the mice were measured and compared. The tumor size in the control group obviously increased from ≈50 to ≈1015 mm^3^; comparably, the tumor growth in the therapeutic group was significantly repressed. The tumor size in the therapeutic group only increased to ≈310 mm^3^ (Figure 7B). Similarly, the average tumor weight in the therapeutic group is relatively lighter than that in the control group (Figure 7C). The weight of the nude mice in the control group had a mild reduction during the later phase of the experiment, which indicated that tumors had already impacted the growth of the nude mice (Figure 7D). These illustrate that NMQDs-Ti_3_C_2_Tx prevents xenograft tumor growth in mice.

At the end of the experiment, tumors were removed from the nude mice. We could intuitively observe black alteration on the tumor xenograft mouse models after NMQDs-Ti_3_C_2_Tx injection, and black regions could be found on the tumor masses (Figure 7E). Through Hematoxylin and eosin (H&E) staining of the tumor tissues, we could clarify that the black region on the tumor masses is a large area of necrosis (Figures 7F,G), which is in agreement with *in vitro* experiments. Necrosis would induce the release of new antigens and activate the immune system, thereby exerting a long-term antitumor effect (Bomze et al., 2019). We also observed a boundary line between necrosis and normal tumor tissue from the therapeutic group (Figure 7H). These illustrate that NMQDs-Ti_3_C_2_Tx shows no toxicity to the tumor cells far away from the injection site. These features of NMQDs-Ti_3_C_2_Tx make it very possible for topical intraocular treatment. However, further study is required to explore the possibility. This is the limitation of the study.

The intraocular biocompatibility of NMQDs-Ti_3_C_2_Tx determines the possibility of future local clinical application. Hence, the *in vivo* intraocular biosafety was investigated on C57BL/6 mice injected with NMQDs-Ti_3_C_2_Tx at 200 μg ml^−1^ intravitreally. After 3 days, optical coherence tomography (OCT) and histochemistry were used to evaluate the morphologic characteristics of the retina. We observed no significant changes through fundus images (Figure 7I). Similarly, the structure of the retina did not reveal noticeable changes compared with the control group (Figure 7J). Additionally, H&E staining disclosed that intraocular treatment with NMQDs-Ti_3_C_2_Tx did not significantly affect the whole retina (Figure 7K). All of these illuminate that NMQDs-Ti_3_C_2_Tx has excellent intraocular biocompatibility.

## 4 Conclusion

In summary, we illustrate that NMQDs-Ti_3_C_2_Tx efficiently inhibits the proliferation, invasion, and migration of UM cells and exerts robust antitumor activity *in vivo*. Furthermore, NMQDs-Ti_3_C_2_Tx exhibits excellent ocular biocompatibility and has no noticeable side effects, providing the possibility for further clinical application of NMQDs-Ti_3_C_2_Tx. Naturally, we hope our work will provide the probability of new clinical development for the topical treatment of UM and expand the prospects of nanoparticles to treat more ocular malignant tumors in the future.

## Data Availability

The raw data supporting the conclusions of this article will be made available by the authors, without undue reservation.
