# Supplementary material for: Highly tumoricidal efficiency of non-oxidized MXene-Ti3C2Tx quantum dots on human uveal melanoma
Source: Front Bioeng Biotechnol. 2022 Oct 6;10:1028470. doi: 10.3389/fbioe.2022.1028470 (PMC9582440; doi:10.3389/fbioe.2022.1028470)
Supplement: Supplementary file 1 [file DataSheet1.docx]

Supplementary Material

# Supplementary Figures and Tables

## Supplementary Figures

**
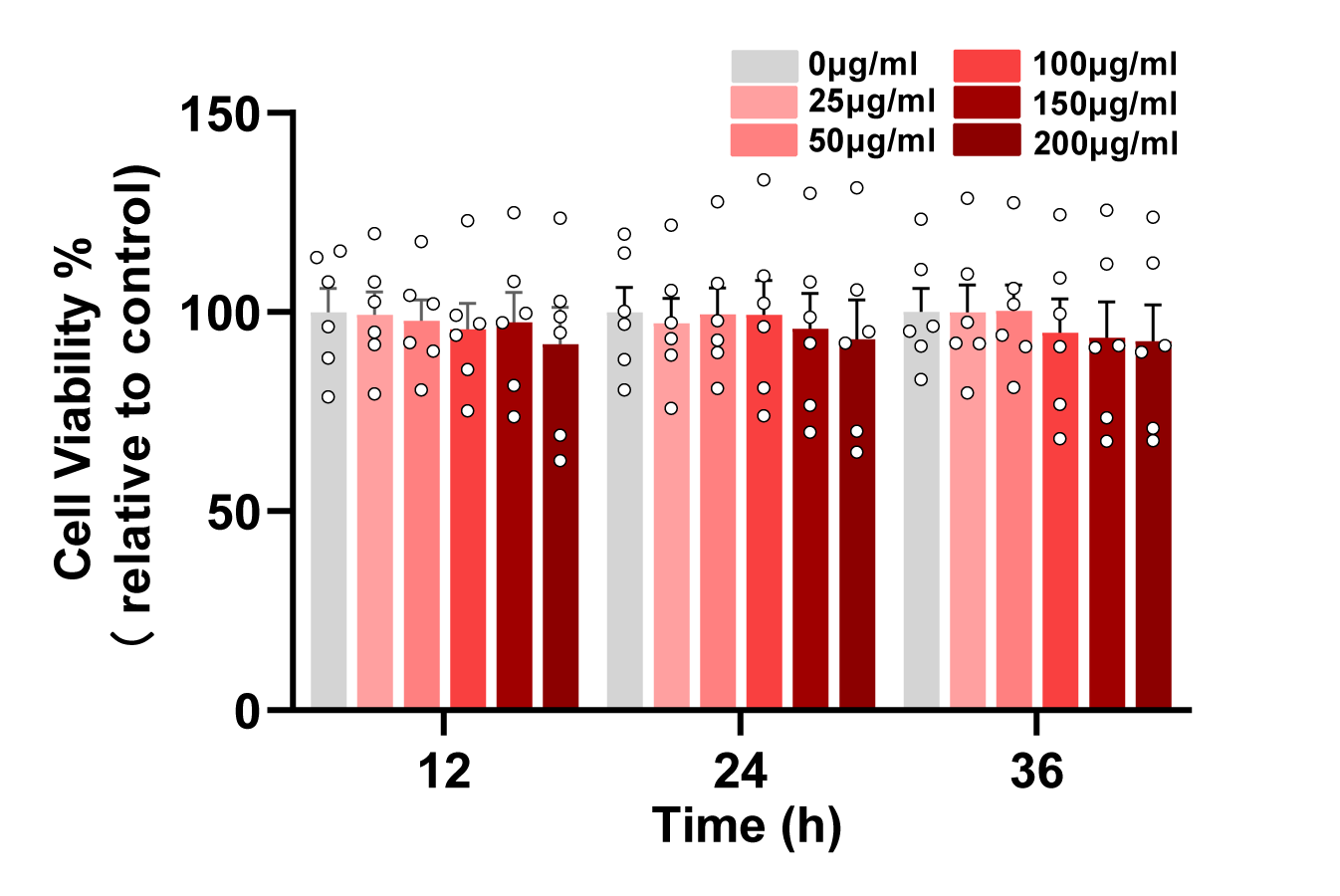
**

**Supplementary Figure 1.** The proliferation of RPE cells was measured by CCK-8 assay. RPE cells were cultured with 0-200μg mL^−1^ NMQDs-Ti_3_C_2_Tx for 12, 24, and 36h. With increasing time in culture, the degree of cell death has not changed significantly with increasing concentrations of NMQDs-Ti_3_C_2_Tx.

**
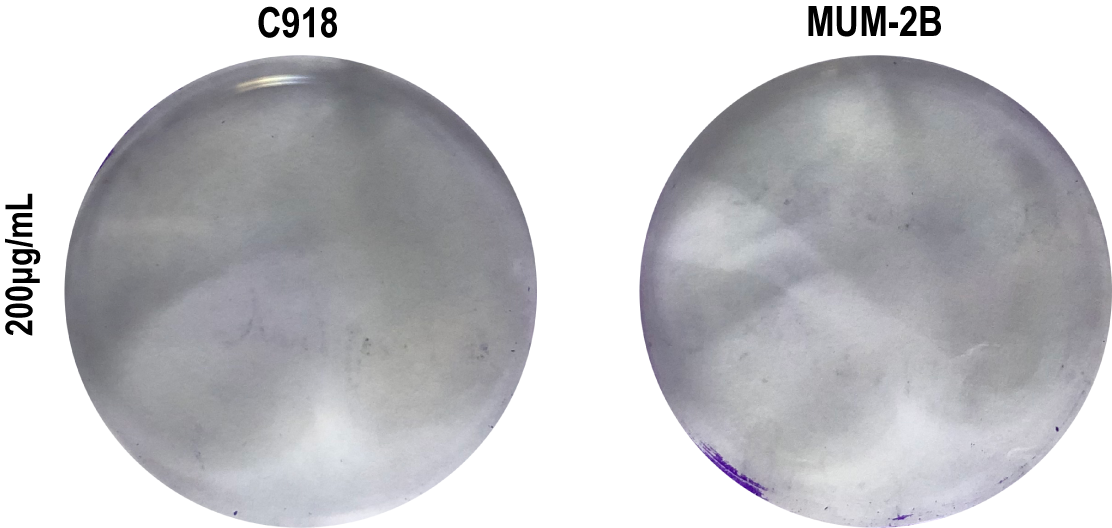
**

**Supplementary Figure 2.** Colony formation assay results. C918 and Mum-2B UM cells were cultured with 200μg mL^−1^ of NMQDs-Ti_3_C_2_Tx for 12 days.

**
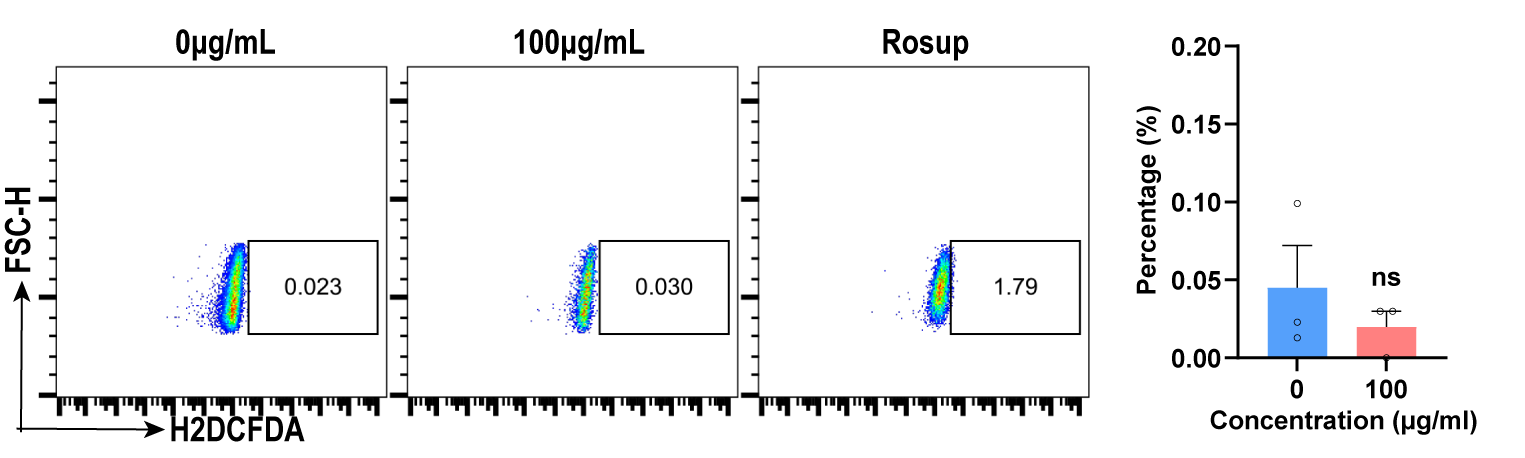
**

**Supplementary Figure 3.** Flow cytometry coupled with H2DCFDA staining was used to determine the levels of ROS in RPE cells treated with 100μg mL^−1^. Rosup was used as a reagent to generate a positive control group with relatively high ROS abundance.

**
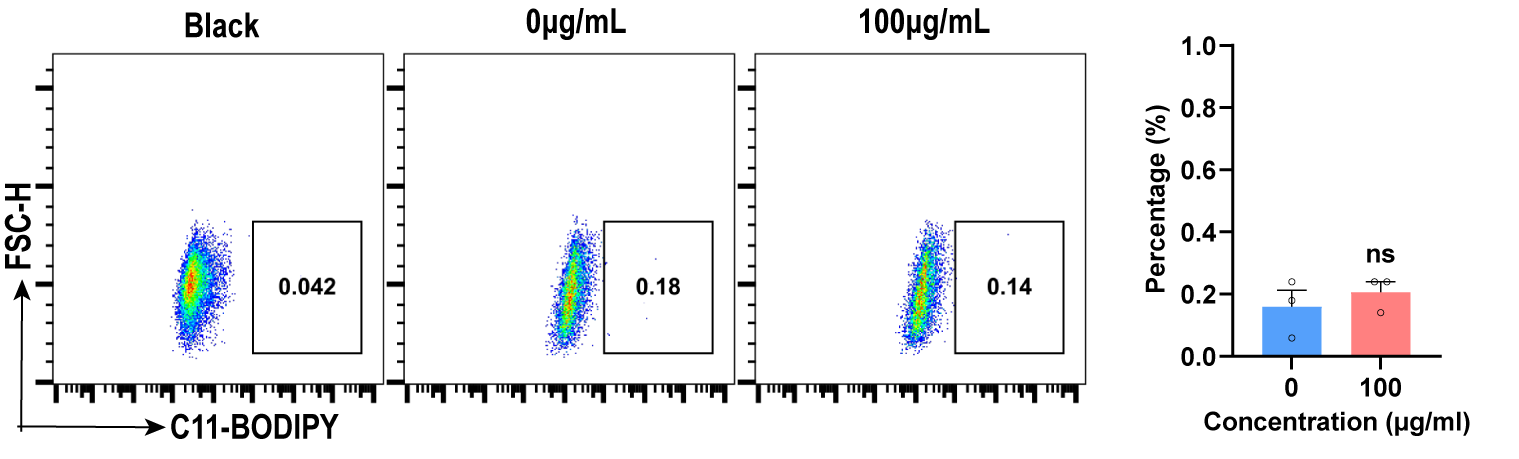
**

**Supplementary Figure 4.** Flow cytometry coupled with C11-BODIPY staining was used to determine levels of lipid peroxides in RPE cells treated with 100μg mL^−1^ NMQDs-Ti_3_C_2_Tx.


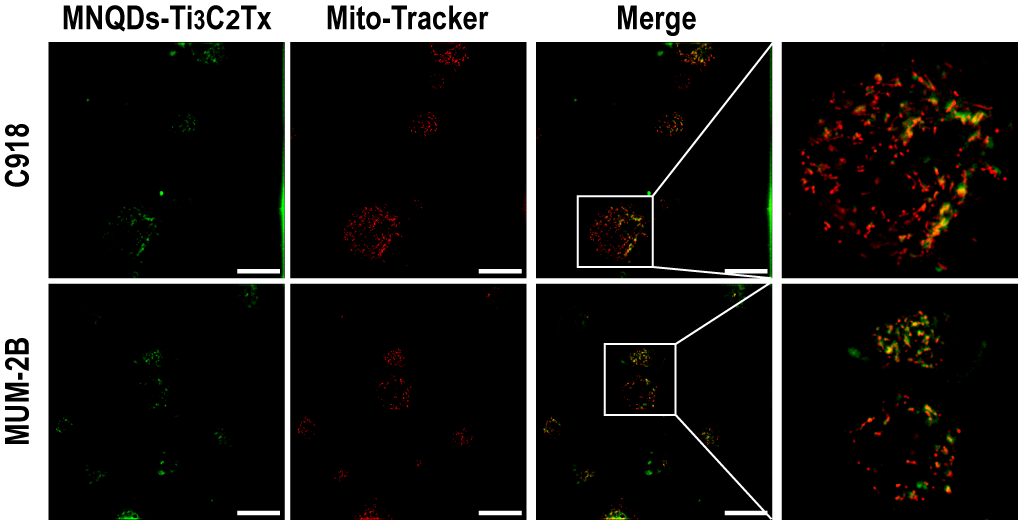


**Supplementary Figure 5.** Colocalization of NMQDs-Ti_3_C_2_Tx and mitochondrion in UM cell lines. Scale bar: 20 μm.


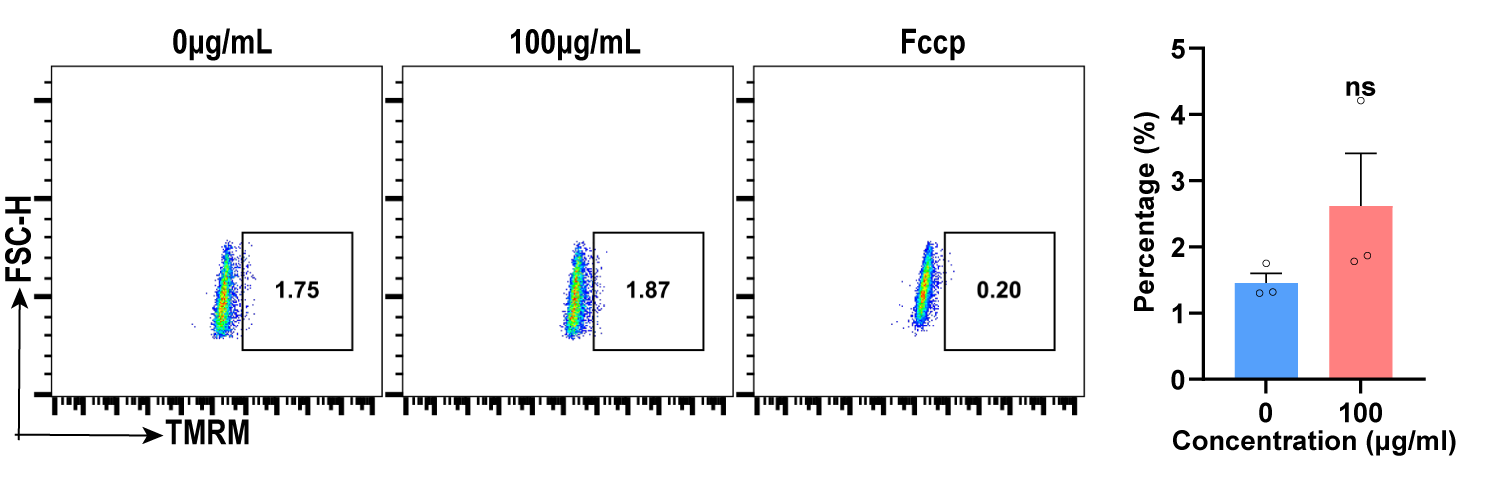


**Supplementary Figure 6.** Flow cytometry coupled with TMRM staining was used to determine the levels of mitochondrial membrane potential（MMP） in RPE cells treated with 100μg mL^−1^. Fccp was used as a reagent to generate a positive control group with relatively low MMP.


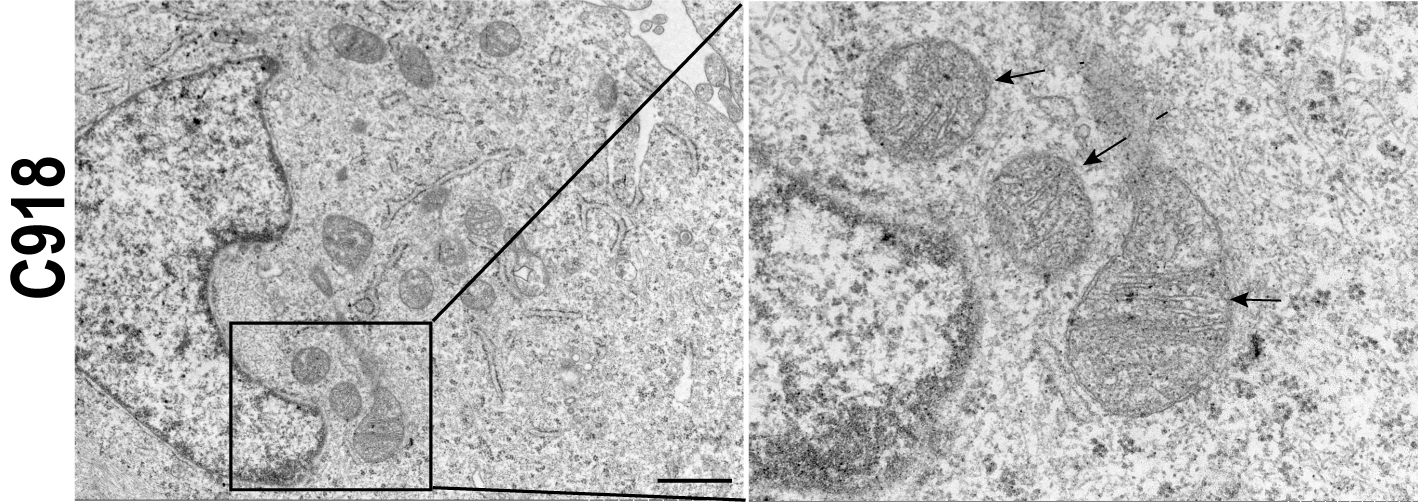


**Supplementary Figure 7.** Bio-TEM image and partial enlarged images of normal C918 cells. The black arrows point to mitochondria. Scale bar: 10 μm.


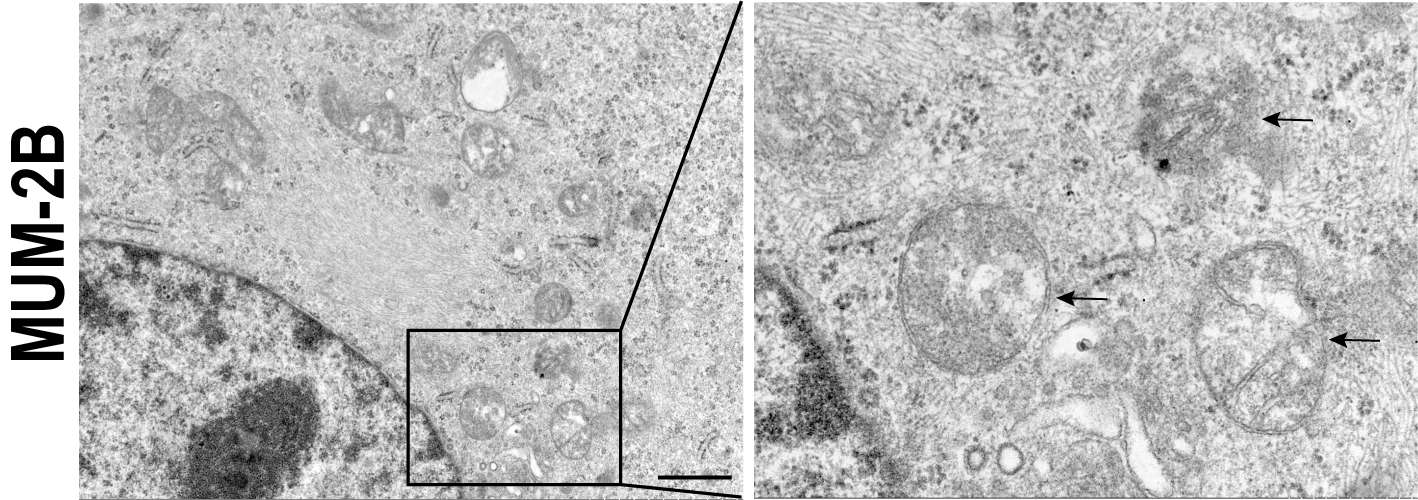


**Supplementary Figure 8.** Bio-TEM image and partial enlarged images of normal MUM-2B cells. The black arrows point to mitochondria. Scale bar: 10 μm.

**
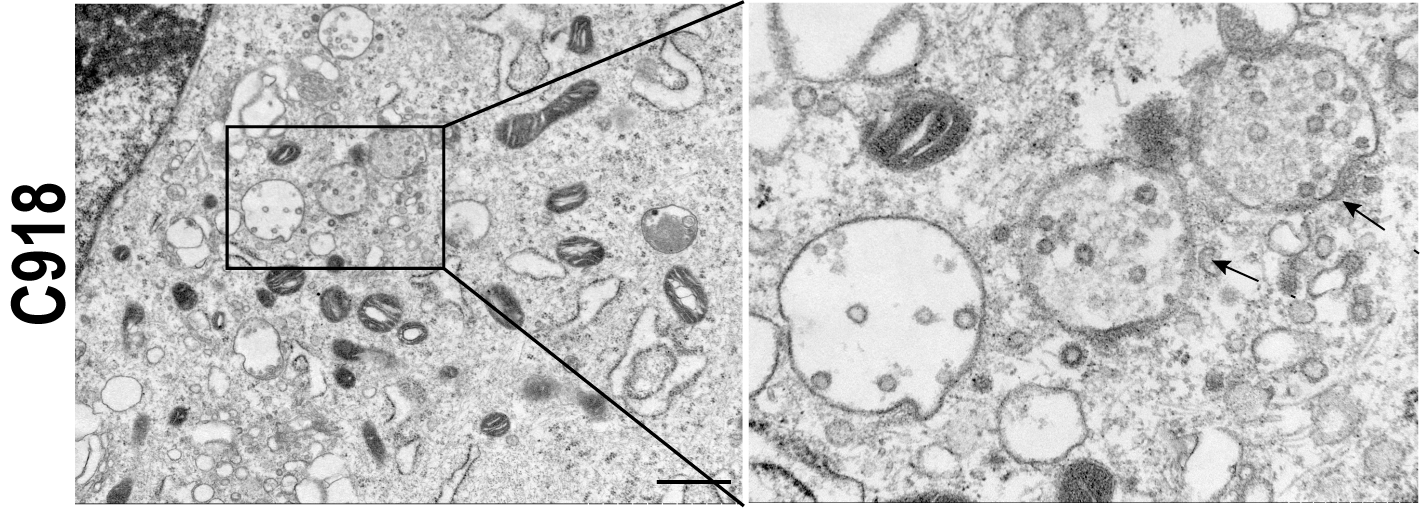
**

**Supplementary Figure 9.** Bio-TEM image and partial enlarged images of normal C918 cells. The black arrows indicated autolysosomes where no nanoparticle materials are seen. Scale bar: 10 μm.

**
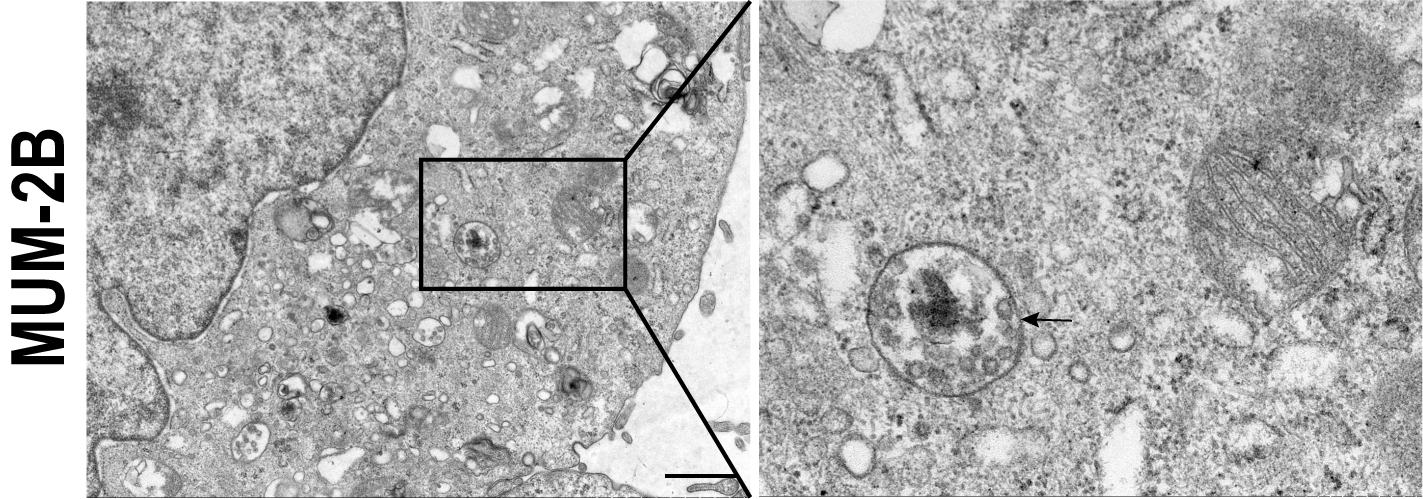
**

**Supplementary Figure 10.** Bio-TEM and partial enlarged images of normal MUM-2B cells. The black arrows indicated autolysosomes where no nanoparticle materials are seen. Scale bar: 10 μm.

## Supplementary Table

| Gen | Forward (5’–3’) | Reverse (5’–3’) |
| --- | --- | --- |
| SLC7A11 | TCTTTGTTGCCCTCTCCTGC | GTAGAGGAGTGTGCTTGCGG |
| PTGS2 | TGTCAAAACCGAGGTGTATGTA | AACGTTCCAAAATCCCTTGAAG |
| LC3 | ATCGCGGACATCTACGAGC | AGGTTTCCTGGGAGGCGTA |
| GAPDH | GCACCGTCAAGGCTGAGAAC | TGGTGAAGACGCCAGTGGA |

**Supplementary Table 1.** Primers used in qRT-PCR.
